# A review of cystic fibrosis: Basic and clinical aspects

**DOI:** 10.1002/ame2.12180

**Published:** 2021-09-16

**Authors:** Qionghua Chen, Yuelin Shen, Jingyang Zheng

**Affiliations:** ^1^ Department No. 2 of Respiratory Medicine Beijing Children’s Hospital Capital Medical University National Center for Children’s Health Beijing China; ^2^ Department of Respiratory Medicine Quanzhou Children’s Hospital Fujian Province Quanzhou China

**Keywords:** animal model, CFTR, Chinese, clinical feature, cystic fibrosis, mutation

## Abstract

Cystic fibrosis is an autosomal recessive disease caused by mutations of the gene encoding the cystic fibrosis transmembrane conductance regulator (CFTR). Here we summarize, at the basic descriptive level, clinical and genetic characteristics of cystic fibrosis gene mutations, while emphasizing differences between CF mutations found in Chinese pediatric CF patients compared to those found in Caucasian CF patients. In addition, we describe animal models used to study human cystic fibrosis disease and highlight unique features of each model that mimic specific human CF‐associated signs and symptoms. At the clinical level, we summarize CF clinical manifestations and diagnostic, treatment, and prognostic methods to provide clinicians with information toward reducing CF misdiagnosis and missed diagnosis rates.

## Graphical abstract

Cystic fibrosis is an autosomal recessive disease caused by mutations of the gene encoding the cystic fibrosis transmembrane conductance regulator (CFTR). We summarize clinical and genetic characteristics of cystic fibrosis gene mutations, as well as animal models used to study human cystic fibrosis disease.

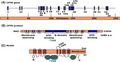

## 1 INTRODUCTION

Cystic fibrosis (CF), a monogenic disease, is the most common life‐shortening autosomal recessive disease that afflicts people of Northern European descent. It was first formally reported to the worldwide medical community in 1949. According to the American Cystic Fibrosis Foundation patient registry, there are currently more than 30 000 CF patients in the United States and more than 70 000 CF patients throughout the world. Globally, about 1000 cases of CF are newly diagnosed every year, with over 75% of CF patients diagnosed at 2 years of age and an average age at diagnosis of about 3 years of age. CF incidence rates vary around the world, but rates as high as 1 in 2000 to 3000 live births are associated with Caucasian populations with Northern European ancestry. The median predicted survival time of CF patients in the United States is approximately 47.4 years (95% CI, 44.2‐50.3) according to the Cystic Fibrosis Foundation 2018 Registry Report. However, epidemiological data on CF prevalence in China have not yet been reported, aside from observations that the genotypic spectrum of Chinese CF varies widely among resident subpopulations based on their geographical and ethnic origins.

Numerous animal CF models have been established based on specific types of human CFTR mutations, but models differ in their effectiveness in mirroring features of human CF‐specific disease. For example, the mouse CF model differs markedly from human CF at the pathological level, while at the molecular level *CFTR* genes of pig and human are highly homologous, but their corresponding CFTR protein structures and functions are vastly different. At present, ferret and rabbit CF models hold promise as human CF models, but additional models based on other species should also be evaluated. Meanwhile, the introduction of human *CFTR* genes harboring CFTR mutations into genomes of animals holds promise as a strategy for creating better animal models for human CF. Nevertheless, current animal models each have their own unique features that are useful for studying particular aspects of human CF disease, as described below.

## 2 PATHOGENESIS OF CYSTIC FIBROSIS

### 2.1 Characteristics of the human cystic fibrosis gene and encoded CFTR protein

Cystic fibrosis is caused by pathogenic mutations in a single large gene located on human chromosome 7 that encodes the cystic fibrosis transmembrane conductance regulator (CFTR) protein.^1^, ^2^, ^3^ CFTR belongs to the ABC (ATP‐binding cassette) family of proteins, a large group of related proteins that share transmembrane transport functions. The *CFTR* gene comprises 250 kilobases of genomic sequence that encodes an epithelial cell protein that is composed of 1480 amino acids in its mature state. The CFTR protein forms a cell membrane‐spanning chloride channel whose function is regulated by phosphorylation mediated by cAMP‐dependent phosphokinases. CFTR phosphorylation in the presence of ATP can trigger channel opening to allow migration of about 10 chloride ions to the outside of the cell every minute. Certain *CFTR* gene mutations lead to generation of defective CFTR proteins that cannot be processed normally by the endoplasmic reticulum for effective transport to the cell membrane. The few mutated CFTR protein molecules that do reach the cell membrane are dysfunctional and thus cannot carry out chloride ion transport, leading to accumulation of chloride ions and associated water molecules in epithelial cells and lack of hydration of extracellular mucus and secretions. The most common pathogenic mutation found in Caucasians of Northern European descent is a deletion mutant designated F508del (with deletion of phenylalanine at site 508 caused by genomic deletion of three nucleotides, designated c.1521_1523delCTT). Indeed, approximately 70% of CF Caucasian patients in the United States harbor this specific *CFTR* mutation, with severe clinical manifestations observed in patients who are homozygous for the delF508 genotype.

The structure of normal CFTR protein contains two groups of six membrane‐spanning structural motifs, two intracellular nucleotide‐binding folds (NBFs), and a highly charged ‘R domain’ containing multiple phosphorylation sites. Activation of the chloride channel requires phosphokinase A‐mediated phosphorylation of the R domain and sustained ATP levels within the NBFs.^4^, ^5^

### 2.2 Genetic mutation types in CFTR

*CFTR* mutations are currently categorized according to cause of dysfunction, including dysfunctional protein translation, cell processing, or CFTR channel gating. Missense (single amino acid substitution) mutations account for 38.74% of CFTR mutants, frameshift (insertion or deletion) mutations account for 16.25%, splicing (incorrect intron splicing) mutations account for 10.93%, and nonsense (early termination codon) mutations account for 8.41% of all known CFTR mutations detected worldwide.^6^

Mutations of the *CFTR* gene fall into six different classes that roughly correspond to specific types of CFTR dysfunction.^7^, ^8^ In general, mutations in classes I to III cause more severe disease than those in classes IV to VI.^8^, ^9^However, clinical manifestations of CF caused by any particular combination of mutations can vary, perhaps due to effects of gene modifiers. For example, genotype‐phenotype correlations are weak for CF associated with pulmonary disease, but are somewhat stronger for CF types associated with pancreatic insufficiency. Indeed, in most cases specific mutations should not be used to make assumptions about CF severity for an individual patient, while clinical decisions should be guided by patient metrics of growth, lung function, and nutritional status. Nevertheless, characterization of mutations may be useful to guide initial therapy for some patients, as several new therapies have been recently developed that target CF disease caused by specific classes of *CFTR* mutations. The five types of CFTR mutations are listed below.

Class I mutations: Defective protein production. This type of defect is usually caused by nonsense, frameshift, or splice‐site mutations, leading to premature termination of messenger RNA (mRNA) transcripts and a complete absence of CFTR protein. Examples include G542X, W1282X, R553X, 621+G>T, and 1717‐1G>A.^10^

Class II mutations: Defective protein processing. This class of mutation causes abnormal post‐translational processing of the CFTR protein, which prevents the protein from trafficking to the correct cellular location, as exemplified by the F508del mutation that is present in a homozygous state in approximately 50% of CF patients and in at least a heterozygous state in 90% of CF patients.^10^

Class III mutations: Defective regulation. These mutations cause diminished channel activity even when ATP levels are adequate. Many mutations alter NBF ATP‐binding regions (designated NBO1 and NBO2), whereby some mutants retain varying degrees of sensitivity to nucleotide binding. The mutation giving rise to CFTR substitution G551D, which abolishes ATP binding, is the most common class III mutation in Caucasian populations. Meanwhile, other CFTR mutations within the region encoding the CFTR R domain may also fall into this category.^10^

Class IV mutations: Defective conduction. CTFR protein is produced and transported correctly to the cell surface. However, the rate of ion flow and the duration of channel opening are reduced as compared to normal CFTR protein even though chloride currents are generated in response to cAMP stimulation. A mutation that induces a CFTR protein amino acid substitution (R117H) is the most common class IV mutation in Caucasian populations.^10^

Class V mutations: Reduced amounts of functional CFTR protein. This class is not included in some classification schemes. It includes several mutations that alter mRNA stability and other types of mutations that alter stability of the mature CFTR protein (with the latter sometimes classified separately into an additional class, class VI).^8^, ^11^

Class VI mutations: Decreased CFTR stability. This class causes substantial plasma membrane instability and includes Phe508del when rescued by most correctors (rPhe508del).^8^

### 2.3 Distribution of CF gene mutation

CF is being increasingly detected in regions where it was previously undiagnosed due to a lack of clinicians with knowledge of the disease, including areas of South and East Asia, Africa, and Latin America. Due to increased newborn CF screening and detection of individuals with mild CF or disease limited to one organ system, CF prevalence rates are likely to rise. To date, 2107 mutations have been entered into the Cystic Fibrosis Mutation Database (www.genet.sickkids.on.ca/StatisticsPage.html) (Table 1).

**TABLE 1:** Statistics by CF mutation type

Mutation type	Count	Frequency %
Missense	815	38.74
Frameshift	342	16.25
Splicing	230	10.93
Nonsense	177	8.41
In frame in/del	43	2.04
Large in/del	59	2.80
Promoter	17	0.81
Sequence variation	269	12.79
Unknown	152	7.22

### 2.4 Characteristics of mutations in Chinese pediatric CF patients

In order to collect information pertaining to CFTR mutations associated with pediatric CF cases in China, we searched scientific literature repositories that included the Chinese Knowledge Infrastructure Digital Library, Wanfang database, VIP database, and PubMed for reports published from January 1, 1975, to June 1, 2021.^12^, ^13^, ^14^, ^15^, ^16^, ^17^, ^18^, ^19^, ^20^, ^21^, ^22^, ^23^, ^24^, ^25^, ^26^, ^27^, ^28^, ^29^, ^30^ Duplicate reports, reports of undiagnosed cases, and reports lacking *CFTR* gene mutation information were excluded from our final analysis. From the final set of reports, we detected 106 Chinese pediatric CF cases associated with 101 *CFTR* gene mutations (Table 2). Our findings revealed that the *CFTR* gene mutation spectrum in Chinese pediatric CF patients differed markedly from the corresponding spectrum for European and American countries. The most common mutation detected in Chinese pediatric CF patients was c.2909G>A, whereas the common mutation c.1766+5G>T found in Chinese CF patients was not found in Caucasians, while the most frequent F508del mutation in Caucasians was rare in China. The majority of mutations in Chinese patients have been found only once or are absent in Caucasians. In the carrier screening panel of CF recommended by the American College of Medical Genetics, the 23 most common CFTR variants cover about 84% of CF‐causing mutations among Caucasians. However, only 4 mutations have been observed in Chinese CF patients. Our results also revealed the value of neonatal screening for achieving early CF detection and treatment in some countries. Moreover, the results also revealed that a CF diagnosis can be confirmed in some cases using sweat chloride‐based tests, while in other cases genetic testing is needed to confirm a CF diagnosis.

**TABLE 2:** *CFTR* gene mutation in Chinese children with cystic fibrosis

Author	Year of publication	Source	Gender	Age of diagnosis	Sputum culture	Severity	Nucleotide change
Wang	1993	Taiwan, China	F	6 m	NA	Severe	c.1766+5G>T,2215insG,G2816A
Zielenskid	1995	Taiwan, China	F	8 mo	*Pa*	Died	c.1766+5G>T Homozygous
Crawford	1995	Portuguese/Chinese Mixed	F	3 y	NA	NA	c.1766+1G>T
Chen B	1995	Mainland China	F	—	NA	NA	E2 del about 30bp
Wu	2000	Mainland, China	F	14 y	NA	Severe	c.1766+5G>T, 2215insG,G2816A;
			M	17 y	NA	Severe	c.1766+5G>T, 2215insG,G2816A
Alper	2003	Chinese/Vietna	M	1.5 y	NA	Mild	G151T,989‐992insA;
		mese mixed					
		Taiwan, China	F	6 mo	NA	Mild	c.1766+5G>T,cis2215insG+G2816A
Chen	2005	Taiwan, China	M	3.42 y	*Pa*	Severe	R553X, homozygous
Li	2006	Mainland China	F	14 y	NA	Mild	699C>A, 3821‐3823delT
Wang	2012	Mainland China	F	14 y	*Pa*	Mild	W679X, homogyzous
Liu J	2012	Mainland China	F,	13.4 y	*Pa*	Mild	2909G>A,263T>G
			F	10 y	*Pa*	Died	3196C>T, homogyzous
Cheng	2012	Mainland China	F	12 y	NA	NA	W679X, 1342‐11TTT>G,3120+2T>C
Liu	2015	Mainland China	M,	12 y	NA	NA	c.95T>C,c.1657C>T
			M,	10 y	NA	NA	c.293A>G,c.558C>G
			M,	16 y	NA	NA	c.2052dupA, △E18‐E20(c.2909‐?_3367 +?del)
			F,	16 y	NA	NA	c.2909G>A, △E7‐E11(c.744‐?_1584 +?del)
			F	10 y	NA	NA	c.1679 + 2T>C, c.2658‐1G>C
Xie	2015	Mainland China	M	12 y	NA	Severe	c.865A>T,c.3651_3652insAAAT
			M	15 y	NA	Died	c.865A>T,c.3651_3652insAAAT
Chu	2016	Mainland China	M	9 y	*Pa*	Died	c.597+2insACAT,c.1766+5G>T
Xu B	2016	Mainland China	F	10 y	*Pa*	Mild	c.595C>T, homozygous
			M	8 mo	*Pa*	Mild	c.595C>T,c.2290C>T
Li L	2016	Mainland China	M	5 mo	Negative	Mild	c.650A>G,c.3406G>A, c.214G>A
Leung	2016	Hongkong, China	M	17 y	*PA, SA,HI, PM,BC*	Died	c.1766+5G>T,c.3068T>G
		Hongkong, China	M,	6 mo	*SA, MC,HI*	Mild	c.1766+5G>T,c.3140‐26A>G
		Hongkong, China	M	2 mo	*SA, Ac, PA,KP, SM,*	Mild	c.868C>T,c.3068T>G
					*NTM*		
	2016	Mainland China	F	9 mo	*PA, SA*	Mild	c.1657C>T,c.3068T>G
		Mainland China	F	13 mo	*Pa*	Mild	c.3068T>G, homozygous
Tian	2016	Mainland China	F	15 y	*Pa*	Mild	c.2909G>A, c.2374C>T
			F	1 y	*Pa, SA*	Severe	c.2909G>A, c.2125C>T
			M	13 y	*Pa*	Mild	c.3700A>G,c.959‐960insA
			M	15 y	*Pa*	Mild	c.3635delT,not detecte
			F	4 y	*Pa*	Mild	c.2909G>A,c.263T>G
			F	13 y	Negative	Mild	c.2909G>A,c.2907A>C
Shen	2016	Mainland China	M	11.58 y	*A fumigatus*	Mild	c.1699G>T,c.3909C>G
			F	10.58 y	*SA*	Mild	c.263T>G,c.1766+5G>T,c.110C>G
			M	13.25 y	*Pa*	Mild	c.3700A>G,c.960_961insA
			F	13.67 y	*Pa*	Mild	c.263T>G,c.2909G>A
			M	7.17 y	*Sm,*	Mild	c.326A>G,c.1000C>T,c.1666A>G
			F	10.67 y	*Pa*, *SA*	Mild	c.595C>T heterozygous
			F	7.75 y	*Pa*, *A fumigatus*	Mild	c.223C>T, c.326A>G
			F	7.33 y	*Kp, SA*	Mild	c.1000C>T heterozygous
			F	10.17 y	*Pa*	Died	c.263T>G, homozygous
			F	11.08 y	*Sp*	Severe	c.1666A>G, heterozygous
			M	8.25 y	*Pa*	Mild	c.293A>G,c.558C>G
			F	4.17 y	*Pa, SA*	Alive	c.326A>G,c.2374C>T
			M	3.67 y	*SA*	Alive	c.1666A>G, homozygous
			F	12.67 y	*Pa*	Mild	c.293A>G heterozygous
			M	11.00 y	*Pa*	Mild	c.648G>A,c.2491‐126T>C
			F	10.33 y	*Pa*	Died	c.3196C>T,c.3196C>T
			M	11.17 y	*Pa, Sa, A fumigatus*	Mild	c.414_415insCTA, homozygous
			F	3.42 y	*Pa*	Died	c.1075C>T,c.3307delA
			F	14 y	*MRSA*	Mild	c.2909G>A, homogyzous
Zheng, Cao	2017	Mainland China	M	5 y	*Pa*	Mild	c.3196C>T,c.870‐1G>C
			F	5 y	*Pa*	Mild	c.3G>A,c.1572C>A
Cai^12^	2017	Mainlan China	F	7 y	NA	NA	c.3197G>A,c.1766+5G>T
Sun Y^13^	2017	Mainland China	M	9 y	*Pa*	NA	c.595C>T homozygous
			F	6 y	*Pa*	NA	c.1117‐1G>C,c.2909G>A
			M	13 y	None	NA	c.4056G>C, heterozygous
			F	2 y	None	NA	c.1666A>G, heterozygous
Wang G^14^	2017	Mainland China	F	10.2 y	NA	Alive	c.263T>G
			F	5.7 y	NA	Alive	c.3G>A,c.1572C>A
			F	7.5 y	NA	Alive	c.532G>A
			F	0.5 y	NA	Alive	c.532G>A
			M	5.8 y	NA	Alive	c.2909G>A,c.3196C>A
Hu X^15^	2017	Mainland China	F	6 y	*Pa*	Mild	c.263T>G
Liu^16^	2017	Mainland China	F	11 y	None	Mild	c.3140‐454_c.3367+249del931ins13
Li Z^17^	2018	Mainland China	M	1 y	*SA*	Mild	c.1432delC,c.1657C>T
Li J^18^	2017	Mainland China	F	16 mo	*Pa*	Mild	c.2125C>T, c.2909G>A
Guo Z^19^	2017	Mainland China	F	9 mo	*Pa*	Mild	c.1373G>A, c.271G>A
Xu^20^	2017	Mainland China	M	9 y	*Pa*	Alive	c.1766+5G>T, c.579+1_c.579+2insACAT
			M	5 y	*Pa*	Alive	c.595C>T
Qiu^21^	2018	Mainland China	F	6 mo	*Pa*	Mild	c.1040G>A, c.4056G>C, c.1526G>C
			M	4 mo	Negative	Mild	c.2909G>A
			F	6 mo	*SA*	Mild	c.1116+1G>A, c.3062C>T
Chen L^22^	2019	Mainland China	F	9 y	*Pa*	Mild	c.1766+5G>T, c.2805delA
Li H^23^	2020	Mainland China	M	4 mo	*Pa*	Mild	c.1766+5G>T, c.1657C>T
			M	13 y	*Pa*	Died	c.1657C>T, c.1408G>A
Wang D^24^	2020	Mainland China	F	6.75 y	*Pa*	Mild	c.2551C>T, c.2834C>T
Li Z^25^	2020	Mainland China	M	7 mo	*Pa*	Mild	c.595C>T, c.95T>G
Shen^26^	2020	Mainland China	F	5 mo	*Pa, SA*	Alive	c.263T>G, c.2909G>A
			M	3 mo	*Pa, SA*		c.2909G>A, ΔE23 (c.3718‐?_3873+?del)
			F	5 mo	*KP, S maltophilia, Sp,*		c.223C>T, c.2909G>A
					*E coli*		
			M	9 mo	*Kp, Pa, SA, MRSA, A baumannii, E coli, A fumigatus,C albicans*		c.262_266delTTATA, c.3859delG
			M	8 mo	*SA*		c.1116+1G>A, c.2909G>A
			F	11 mo	*Pa, A baumannii*		c.2236_2246delGAGGCGATACTinsAAAAATC,c.3635delT
			F	26 mo	*Pa, SA*		c.1521_1523delCTT homozygous
			M	56 mo	*Pa, SA*		c.1210‐3C>G, c.3964‐7A>G
			F	100 mo	*B cenocepacia, Pa.SA*		c.1000C>T, c.1733T>C
					*A fumigatus, A flavus, C albicans*		
			F	106 mo	*Pa, SA, A flavus, C albicans*		c.579+2insACAT, c.1766+5G>T
			M	9 mo	None		c.3068T>G, c.595C>T
Shen Y^27^	2020	Mainland China	NA	NA	NA	NA	c.3209G>C, c.2328_2329insA
							c.1344‐1347delAGAA, c.2909G>A
							c.1766+5G>T, c.3483C>T
							c.595C>T, c.2290C>T

							c.2834C>T
							c.350G>A, c.2036G>A
							c.2909G>A, c.1000C>T


							c.3841C>T, c.298C>T
							c.293A>G, c.3068T>G
Li L^28^	2021	Mainland China	F	8 y	None	Mild	c.650A>G, c.1231A>G
Li M^29^	2021	Mainland China	F	11 y	*Pa*	Mild	c.2909G>A, c.133T>A, c.125delT
			M	7 y	*Pa*	Mild	c.380T>G
Wang F^30^	2021	Mainland China	M	11 y	*Pa*	Mild	c.1369G>C, c.320C>A

Abbreviations: M, male; F, female; NA, not applicable; *Pa*, *Pseudomonas aeruginosa*; *SA*, *Staphylococcus aureus*; *MRSA*, methicillin‐resistant *Staphylococcus aureus*; *Kp*, *Klebsiella pneumoniae*; Sp, *Streptococcus pneumoniae*; *Ac*, Acinetobacter; *BC*, *Burkholderia cepacia*; *HI*, *Haemophilus influenzae*; *MC*, *Moraxella catarrhalis*; *NTM*, non‐tuberculosis mycobacterium; *PM*, *Pseudomonas pseudomallei*; *SM*, *Stenotrophomonas maltophilia*.

## 3 CLINICAL FEATURES OF CYSTIC FIBROSIS

CF is caused by dysfunctional transport of chloride and/or other ions (such as sodium and bicarbonate) that leads to generation of thick, viscous secretions (eg mucus) in the lungs, pancreas, liver, intestine, and reproductive tract and increased salt content in sweat gland secretions. Ultimately, progressive lung disease is the main cause of CF complications and patient mortality.^8^ The course of disease varies greatly and can begin from a few months after birth to decades after birth, with many patients exhibiting mild or atypical symptoms. Therefore, clinicians should take care to avoid excluding CF as a possible diagnosis in cases where patients exhibit only a few typical CF signs and symptoms.

### 3.1 Respiratory tract involvement

Typical respiratory manifestations of CF include a persistent productive cough, hyperinflation of lung fields on chest radiograph, and pulmonary function test findings indicative of obstructive airway disease. As the disease progresses, repeated infections associated with inflammatory cell accumulation and release of cell contents damage bronchial walls, leading to loss of bronchial cartilaginous support and muscle tone and eventual bronchiectasis. Disease progression includes acute exacerbations of cough, tachypnea, dyspnea, increased sputum production, malaise, anorexia, and weight loss. These acute events are associated with acute, transient loss of lung function that improves with treatment but that often progresses to permanent loss of lung function over time.

Although CF patients often vary, transient airway infection with pathogenic bacteria often first occurs early in life. After years of CF disease, chronic airway infection with either *Staphylococcus aureus* or *Pseudomonas aeruginosa* often becomes established and is often detected based on radiographic evidence of bronchiectasis. In addition, airways of CF patients can be colonized or infected by other species of microbes, including *Stenotrophomonas maltophilia*, *Achromobacter xylosoxidans*, *Burkholderia cepacia* complex, nontuberculous mycobacteria (especially *Mycobacterium avium* complex and *Mycobacterium abscessus*), and the filamentous fungus *Aspergillus fumigatus*.^31^ Continuous airway colonisation and infection by bacteria (especially *P aeruginosa*) can enhance the inflammatory response by triggering neutrophils to release large amounts of DNA and matrix proteins into airways. These substances, coupled with CF‐induced impaired airway clearance functions and chronic inflammation, increase airway mucus viscosity. Current research efforts are underway to identify additional bacterial species in CF patient airways, including obligate anaerobes that may be identified using next‐generation sequencing technology.^32^, ^33^

### 3.2 Sinus disease

The majority of CF patients develop sinus disease.^34^ Sinus disease can present with chronic nasal congestion, headaches, cough caused by chronic postnasal drip, and sleep disturbances. Sinus infections can trigger lower respiratory exacerbations in some patients, although organisms found in sinuses do not always match those recovered from lungs. Meanwhile, some individuals with isolated chronic rhinosinusitis have signs and symptoms suggestive of CFTR dysfunction that do not satisfy CF diagnostic criteria, prompting clinicians to refer to this affliction as CFTR‐related disorder. Notably, in one case‐control study, the single *CFTR* mutation rate for a group of chronic rhinosinusitis cases was significantly higher than the corresponding rate for the general population (7% versus 2%).^35^

### 3.3 Digestive system diseases

Approximately two‐thirds of CF patients exhibit CF insufficiency of the exocrine pancreas from birth, with an additional 20% to 25% developing this condition during the first several years of life, and most exhibiting signs of fat malabsorption by one year of age.^36^ CF‐associated pancreatic disease tends to be progressive; many patients with apparently normal or marginal pancreatic function at birth develop overt evidence of pancreatic insufficiency in childhood or adulthood. Overall, approximately 85% of individuals with CF eventually develop clinically significant pancreatic insufficiency.^37^ Common symptoms and signs of pancreatic insufficiency include steatorrhea, characterized by frequent, bulky, foul‐smelling stools that may be oily, as well as failure to thrive or poor weight gain resulting from malabsorption of fat and protein. Infants with severe untreated pancreatic insufficiency occasionally present with edema, hypoproteinemia, electrolyte loss, and anemia due to malabsorption of macro‐ and micronutrients. Some patients also may present with symptoms caused by deficiencies of the fat‐soluble vitamins A, D, E, and K. Vitamin K deficiency can present as a coagulopathy and vitamin D deficiency as rickets. Continued defective ductular and acinar pancreatic secretion functions lead to progressive pancreatic damage that can trigger acute or recurrent pancreatitis. Moreover, patients with exocrine pancreatic insufficiency often develop dysfunction of the endocrine pancreas, leading to glucose intolerance and CF‐related diabetes.

With regard to other CF‐associated digestive system disorders, 10% to 20% of newborns with CF present with meconium ileus characterized by obstruction of the bowel by meconium, which is a risk factor for poor CF prognosis.^38^ Rectal prolapse, which previously was rarely detected in children with CF, has been detected frequently in recent years and appears to be associated with constipation and/or malnutrition. Focal biliary cirrhosis caused by inspissated bile is present in many patients and may cause elevated serum alkaline phosphatase and lobular hepatomegaly. A minority of CF patients develop periportal fibrosis, cirrhosis, symptomatic portal hypertension, and variceal bleeding that are associated with progressive liver disease.^37^

### 3.4 Reproductive system diseases

More than 95% of men with CF are infertile because of defects in sperm transport, although spermatogenesis is not affected. Intriguingly, nearly one‐half of all men with congenital bilateral absence of the vas deferens and normal lung function possess two *CFTR* mutations.^39^ Meanwhile, females with CF are less fertile than normal healthy women, due to malnutrition and the production of abnormally tenacious cervical mucus. Nonetheless, females with CF may become pregnant and those who do should be counselled accordingly about contraception and childbearing decisions.^40^ Indeed, comprehensive genetic counselling is essential for prospective parents with CF.

### 3.5 Nutrition and growth disorders

Patients with CF have reduced bone mineral content and increased rates of fractures and kyphoscoliosis. In all age groups, up to 30% of patients present with clinically significantly reduced bone density, while this proportion approaches 75% in adults with CF.^41^, ^42^ Clubbed fingers (and toes) and hypertrophic osteoarthropathy can also occur in patients, with clubbing of fingers (and toes) found commonly in patients with long‐term disease, while hypertrophic osteoarthropathy is only rarely observed.

## 4 DIAGNOSIS OF CYSTIC FIBROSIS

### 4.1 Diagnostic criteria of cystic fibrosis

Both of the following criteria must be met to diagnose CF^43^: (1) Clinical symptoms consistent with CF in at least one organ system, or a positive newborn CF screening result, or a sibling with CF. (2) Evidence of cystic fibrosis transmembrane conductance regulator (CFTR) dysfunction (any of the following): Elevated sweat chloride ≥60 mmol/L, or presence of two disease‐causing mutations in the *CFTR* gene (one from each parental allele), or abnormal nasal potential difference (NPD) result.

### 4.2 Sweat chloride

The sweat chloride test remains the primary test used for CF diagnosis. If the concentration of chlorine is greater than 60 mmol/L, the diagnosis of CF is confirmed, while a high concentration of 40‐60 mmol/L is suspicious, and a concentration <40 mmol/L is normal (excluding adrenal insufficiency). However, new clinical guidelines^43^ indicate that a sweat chloride concentration <30mmol/L is the normal threshold for all age groups (excluding adrenal insufficiency). Importantly, a normal sweat chloride concentration is observed in approximately 1 percent of CF patients with unusual genotypes, such as the c.3717+12191C>T (legacy name: 3849 + 10 kb C‐T) or poly‐T defects.^43^

### 4.3 CF diagnostic challenges

Due to the wide range of clinical phenotypic differences among CF patients, CF can be difficult to diagnose. Apart from the respiratory manifestation, symptoms involving other organs in Chinese CF patients are not as common as in Caucasians, and in particular there are fewer digestive symptoms. This is true for approximately 10%‐15% of CF patients who have mild symptoms, normal pancreatic function, good nutrition, slow decline of lung function, no obvious family history, borderline normal sweat test results, and only one detected *CFTR* gene‐associated mutation. In addition, if onset of CF is delayed or if the patient has a CF‐associated mutation that is very rare and thus is not included in the scope of routine screening, diagnosing the disease is even more challenging, causing diagnostic delay. At the current time, nearly 70% of typical CF patients are diagnosed before they reach 1 year of age. The median age of CF diagnosis in the United States in 2018 was 3 years of age, while 8% of patients were diagnosed after 10 years of age.^44^ With regard to Chinese CF patients, Shen reported a 5.7‐year delay between the first clinical presentation and the eventual CF diagnosis,^45^ while another study of pediatric Chinese patients revealed a median age at diagnosis of 8 years of age.^46^ Moreover, Chinese CF patients are more likely to have a negative family history, possibly due to the previous one‐child policy. Diagnosis of CF may be less suppressed following the recent implementation of the three‐child policy.

## 5 ANIMAL MODELS OF CYSTIC FIBROSIS

To date, many animal models of CF have been established that vary according to type of CFTR mutation. Phenotypes of human and animal models of cystic fibrosis are listed in Table 3.

**TABLE 3:** Phenotypes of human and animal cystic fibrosis models

Species	Spontaneous pulmonary infection	Pancreatic insufficiency	Intestinal diseases	Hepatobiliary diseases	Reproduction
Human	Yes	Yes	Meconium ileus	Biliary cirrhosis	Severe vas deferens defect
Mouse	No	No	Meconium ileus, Fetal	No	Female fertility decline
Pig	Yes	Yes	100% Meconium ileus	Biliary cirrhosis	Severe vas deferens defect
Ferret	Yes	Yes	75% Meconium ileus	Liver diseases	Severe vas deferens defect

### 5.1 Murine models of CF

Murine CF models have been developed, but they do not mirror human disease very well due to differences in lung and pancreas anatomical structures and physiologies between mice and humans. For example, mice with dysfunctional CFTR can present with impaired chloride transport in some types of epithelial cells, but the resulting pathology differs markedly from human CF pathology. By contrast, CF mice harboring the most severe *CFTR* mutation may exhibit gastrointestinal pathological effects resembling those associated with human CF, including intestinal obstruction, mucus accumulation, goblet cell proliferation, and fat absorption disorder. However, most of these mice die of intestinal obstruction soon after birth if they are not fed a special diet, making use of CF mice model impractical as a human CF model. CF mice also exhibit male sterility, as occurs in human male CF patients. Paradoxically, no CF mice exhibit pancreatic insufficiency, possibly due to low *CFTR* gene expression in mouse pancreas, as well as other mechanisms unique to mice, including intracellular calcium‐based activation pathways that enable mouse cells to expel water. Moreover, bacterial infections, inflammatory reactions, mucus accumulation, and tissue remodeling have never been detected within CF mice lungs, although the reasons for the absence of lung pathologies are unknown. One possible factor may be related to the absence of ciliated epithelium and submucosal glands in mouse lung compared to human lung. Another possible factor is the presence in mice of other chloride channels, such as ICACCS, which may compensate for CFTR dysfunction; this potential backup system is absent in human CF patients. In the meantime, modifications of the mouse CF model have been conducted to create better CF mouse models. For example, electrophysiological analysis of CF mouse nasal epithelium has revealed increased sodium current in nasal epithelium. Inhibition of this current via treatment of CF mice with amiloride (an agent that blocks cAMP‐regulated chloride transport) generated a CF mouse phenotype mirroring human CF. Nevertheless, this CF mouse model did not develop lung lesions and was not susceptible to spontaneous pulmonary infections that occur in human CF patients.^47^ Taken together, these results highlight differences between humans and mice with regard to the effects of CFTR dysfunction that may reflect species differences in airway cell biological characteristics, numbers of submucous glands, and expression and activation of other chloride channels. Moreover, even though a *CTFR* gene knockout mouse model has been established, results of studies using this model have not yet been reported. This lack of reporting may reflect the fact that the model did not perform well, since CF mice can utilize a CFTR‐independent alternative Cl^−^ channel that enables cells of CFTR‐deficient mice to secrete Cl^−^ to compensate for the lack of a functional CFTR. Notably, ATP12A belongs to the P2‐type ATPase family and shares sequence homologies with both the gastric H,K‐ATPase (ATP4A) and the Na,K‐ATPase (ATP1A). ATP12A mediates the electroneutral exchange of H^+^ for potassium (K^+^) but may also function in a Na^+^/K^+^ exchange mode.^48^ In humans lacking CFTR, unchecked H^+^ secretion by the nongastric H^+^/K^+^ adenosine triphosphatase (ATP12A) acidified airway surface liquid, which impaired airway host defenses. However, the expression of ATP12A is low in murine airways, which may partly explain the very mild pulmonary phenotype in murine models of CF.^49^ In any case, mouse models are not helpful for studying the long‐term pathology of human CF disease, due to the short lifespan of mice.

### 5.2 CF rat models

Compared with mice, rats are appreciably bigger and provide better tissue specimens and blood samples for analysis. Compared with larger animals, rats have a shorter gestation and earlier sexual maturity. Like humans, rats have extensive submucosal glands, which are implicated in the development of CF airway disease.^50^

To date, several CF rat models have been generated with interesting phenotypes. The first CF rat model was a CFTR‐knockout rat strain.^51^ Recently, two CF rat models of KO and F508del CFTR using CRISPR/Cas9 gene editing have showed encouraging results.^52^, ^53^ They revealed CF manifestations including reduced survival, intestinal obstruction, bioelectric defects in the nasal epithelium, histopathological changes, and male reproductive abnormalities. Moreover, they represent a novel resource to advance the development of CF therapeutics.

### 5.3 Porcine CF models

Pigs have a large number of offspring, mature rapidly, and have a long lifespan, enabling researchers to study long‐term pathology and prognosis of CF. In addition, pig anatomy and physiology mimic corresponding human characteristics and porcine CFTR is 92% homologous in nucleotide sequence to human CFTR. In 2008, Rogers et al^54^ generated *CFTR* gene knockout and delF508 pig models using a recombinant adeno‐associated virus (AAV mediated) method. Lack of functional CFTR protein in CFTR^−/−^ piglets led to similar phenotypic effects in lung, liver, pancreas, and gastrointestinal tract tissues as those found in human CF.^55^, ^56^, ^57^ Meconium ileus was present in 100% of CFTR−/− piglets (compared with 15% in CF infants), which in humans can be fatal without early surgical intervention,^56^ thus limiting potential use of porcine models in some research settings due to risks associated with surgery (including intestinal atresia). Finally, although *CFTR* genes of pigs and humans are similar at the nucleotide level, their encoded CFTR proteins differ markedly in structure and function.

### 5.4 The ferret CF model

The ferret CF model shares many CF pathological characteristics with human CF, especially in newborns. However, considerable effort is needed to produce enough CFTR−/− ferrets to ensure that some animals overcome gastrointestinal pathology and reach puberty so they can be used to model human CF based on similarities of lung pathology. Notably, characteristics of pulmonary infections in CFTR−/− ferrets at the beginning and end of life mirror corresponding pulmonary features associated with human CF, highlighting the potential benefits of this model. Nevertheless, further development of the delF508 and G551D CFTR mutant forms of the ferret CF model are needed to better model human CF.

### 5.5 The rabbit CF model

Recently, CRISPR/CAS9 has been used to generate CFTR knockout and F508del genomic mutations to create CF rabbit models.^58^ Rabbits are considered to be an ideal species for simulating human CF lung disease, as their airway anatomy and inflammatory responses resemble corresponding human characteristics. Preliminary findings of experiments using CF rabbits to model human CF indicate that CF rabbits will likely be useful for modelling human CF disease.

### 5.6 Prospective animal models for CF research

With the development of rapid and accurate gene editing technologies such as CRISPR/CAS9, better animal models of human CF can now be created by introducing specific *CFTR* genes and mutations into animal genomes. Development of animal models that accurately mimic human CF will facilitate development of experimental pulmonary therapies, identification of new therapeutic targets, and enable clarification of complex mechanisms underlying initiation and progression of pulmonary CF.^59^ Although no perfect CF animal model exists, each animal model has its own unique advantages for use in studying specific CF‐related pathogenic mechanisms.

## 6 IMPORTANCE OF ANALYZING CF GENOTYPES

The spectrum of *CFTR* genotypes of Chinese children with CF significantly differs from that of children in European and American countries. The most common CF mutation in Caucasian patients (F508del) is rare in China, while *CFTR* genotypes detected in Chinese CF patients are more diverse than genotypes of Caucasian CF patients.

To help patients, family members, healthcare providers, and scientists understand the complexity and clinical significance of widely recognized CFTR mutations, a team at Johns Hopkins University has developed a website to provide information regarding specific cystic fibrosis mutations (http://www.cftr2.org). By analyzing registered functional data obtained from cellular research studies, researchers can obtain useful information about genotype‐phenotype relationships, especially for individuals with borderline functional CTFR mutations and less severe CF phenotypes.

Although the abovementioned cftr2 website is a valuable tool for studying diagnostic characteristics of patients with CFTR mutations, it cannot replace clinical observations and professional knowledge. Indeed, the use of such a tool may lead to missed diagnoses, especially if the cftr2 database is biased toward common mutation sites in one population, as indicated in new cystic fibrosis guidelines.^43^ Importantly, even though the incidence rate of CF in the Chinese population is lower than the CF incidence rate in Caucasians, the absolute number of mutant alleles in Chinese populations may be quite large due to the presence of a large number of undiagnosed CF patients in China.^60^ Therefore, whole gene sequencing is advocated in China as an effective method for detecting rare or new *CTFR* mutations and decreasing the missed diagnosis rate there.

A major challenge for treatment of CF, a rare disease, is that more than 1000 rare mutations have been detected that are each likely carried by no more than five carriers throughout the world. Thus, traditional clinical methods are not suitable for detecting these rare mutations, warranting use of newer methodologies for diagnosing CF in such cases. Nevertheless, it is important to study rare CF mutations, which can reveal important information about disease prognosis and genotype‐phenotype relationships to better guide patient care and improve patient outcomes. In this era of personalized medicine and treatment, such studies will be extremely important for improving care of patients with rare mutations.

## 7 TREATMENT AND MANAGEMENT OF CYSTIC FIBROSIS

Treatment regimens for CF should be evaluated, improved, and administered in combination with close monitoring to achieve early, active intervention to manage CF. In order to achieve these goals, prospective CF patients should be hospitalized until additional test results and other findings are obtained to support or exclude a CF diagnosis. Once a CF diagnosis has been made, clinicians should immediate initiate patient treatment and educate patients and their families to effectively manage the disease.

### 7.1 CF treatment regimens

Treatment for CF lung disease includes administration of mucus thinner, airway clearance, and antibiotics. To thin mucus, inhalation therapy consisting of hypertonic saline is administered to hydrate thick mucus within CE patient airways. The high osmotic pressure of the solution draws water out of airway epithelial cells to reconstruct the water‐containing surface layer that is absent in CF patients. To address retention of purulent secretions in CF patients that obstruct airflow and damage airways, chest physiotherapy based on postural drainage and percussion is the standard method for clearing secretions, with bronchoscopic lavage also used for this purpose. Although antibiotics are essential for treatment of chronic infections and acute CF exacerbations, long‐term oral antibiotics are generally not recommended for controlling infection. However, long‐term azithromycin use is recommended for many CF patients, due to its anti‐inflammatory and/or antibacterial properties, while long‐term treatments with aerosolized antibiotics against *P aeruginosa* (eg tobramycin and aztreonam) are recommended due to their beneficial effects on lung function. Meanwhile, bronchodilator use has been evaluated for CF treatment in many studies, but such treatment does not appear to benefit CF patients.

Notably, the main CF airway pathological feature is severe neutrophil inflammation. In past years, inflammation was considered necessary for preventing spread of infection, but accumulating evidence suggests that excessive inflammation is generally harmful. Thus, in clinical practice, azithromycin is recommended for all CF patients older than 6 years of age with clinical evidence of airway inflammation (eg chronic cough) or any decrease in FEV1, regardless of the status of *P aeruginosa* infection.

To further support CF patient growth and nutrition, patient diets should be supplemented with pancreatic enzymes, calories, and fat‐soluble vitamins. At present, several new types of drugs^61^, ^62^, ^63^ are under development, of which some are well‐tolerated by patients, including drugs to restore normal function of defective CFTR protein and drugs that have a direct impact on mucociliary clearance. CFTR modulators, such as ivacaftor (Kalydeco), lumacaftor/ivacaftor (Orkambi), tezacaftor/ivacaftor (Symdeko), target different potential CFTR protein defects caused by different gene mutations, thus rendering these drugs effective only for people with specific mutations. Trikafta (tezacaftor plus elexacaftor and ivacaftor) is the third drug approved by FDA that rescues defects caused by F508del, which is superior to its predecessors. Trikafta is also effective in CF patients with one copy of F508del‐CFTR mutation. It demonstrates safety and sustained efficacy for 24 weeks or longer in people with CF and one or more F508del alleles.^64^ To prevent long‐term infection and inflammation that eventually cause irreversible bronchiectasis and respiratory failure, lung transplantation is feasible for end‐stage patient treatment depending on the health of the particular patient.

### 7.2 Management of cystic fibrosis

In addition to timely diagnosis and treatment, long‐term follow‐up and monitoring of CF patient status are also very important. For children, long‐term nutritional assessment should be carried out, including monitoring of height, weight, body mass index, and other indicators, as well as timely nutritional guidance. Moreover, CF patients should receive all recommended routine childhood immunisations, especially the seasonal influenza vaccine. Furthermore, timely evaluation of exacerbations, treatment of severe infections, and maintenance of follow‐up care should all be implemented as necessary components of optimal CF management. Nevertheless, CF chronicity and complexity can seriously impact the mental health of patients and their caregivers by triggering anxiety and depression that can adversely affect treatment compliance and long‐term prognosis. Therefore, psychological counselling is also very important.

## 8 PROGNOSIS OF CYSTIC FIBROSIS

Although cystic fibrosis is currently incurable and greatly reduces life expectancy, the average CF survival age has increased significantly over the past 50 years and now exceeds 40 years of age. Thus, CF is no longer viewed solely as a childhood disease, but now is recognized as a disease of children and adults. Currently more than half of CF patients are adults as old as 60 years of age, indicating that active treatment can improve prognosis, increase quality of life, and prolong lifespan. Time to diagnosis and treatment, severity of lung disease, nutritional and general conditions, and mental state are key factors that influence prognosis.^44^ With regard to pediatric CF patients, attention should be paid to improving awareness and compliance of family members to prevent infection, actively treat acute exacerbations, and comply with recommended care instructions to maximize quality of life and long‐term survival.

## CONFLICT OF INTEREST

The authors have no conflict of interest.
